# DRESS is a cause of increased serum procalcitonin level without bacterial infection

**DOI:** 10.1186/2045-7022-4-S3-P94

**Published:** 2014-07-18

**Authors:** Benoit Ben Said, Frederic Berard, Jean Francois Nicolas

**Affiliations:** 1 Chu Lyon Sud , Inserm U111, Universite Lyon 1, France; 2 Chu Lyon Sud , Inserm U111, Universite Lyon 1, Clinical Immunology and Allergology Department, France

## 

Procalcitonin (PCT) is a protein, which has been initially described in 1993 as specific marker of bacterial infection . PCT has been reported to be useful in the diagnosis and the prognosis evaluation during sepsis or septic shock. PCT adds diagnostic value to the other infection biomarkers as CRP or WBC counts. A negative value of PCT rules out the presence of infections, which is helpful when managing antibiotics therapy. Non bacterial elevations of PCT secondary to inflammatory situation as severe burns are well known. Drug Reaction With Eosinophilia and Systemic Symptoms (DRESS) is one of the most severe drug delayed allergic reaction, characterised by extensive rash, eosinophilia and frequent visceral involvement, with an overall estimated mortality of 10% and high risk sequelae of 11, 5%(5). DRESS often mimics clinical sepsis signs with hypotension, lactic acidosis, multivisceral failure associated to high fever which may lead to inappropriate antibiotics treatment. Thus, making differential diagnosis between DRESS and infection may be every difficult and may delay the prescription of corticosteroids which is the best treatment of visceral involvement in DRESS. Since we observed elevated serum PCT levels in patients with DRESS without any concomitant infection process, we performed a prospective study in 12 consecutive DRESS patients. We demonstrate that PCT is elevated during DRESS (range 2 to 180 ng/l N<0,15)) in patients without any bacterial infection. This led to misdiagnosis and delay in the proper management of DRESS in all the 12 patients of this study. PCT level became normal under systemic corticosteroids in all cases without antibiotics treatment. Interestingly, our results also suggest that an elevated PCT could have a prognosis value since the PCT serum levels correlated with the severity of DRESS during the acute phase and in the follow-up with higher frequency of long term sequelae (5/8 patients with favorable evolution have organ failure sequelae, 4/12 patients have faatal evolution) .PCT was also elevated before the development of organ failure in 6/7 cases when data are available. In conclusion, clinicians should be aware that an increased PCT serum level may be a biological diagnosis marker of DRESS necessitating a specific treatment. More studies are needed to confirm the severity prognosis value of PCT in DRESS.

